# In Vitro Decoated Seed Germination and Seedling Development for Propagation of Wild Mandrake (*Mandragora autumnalis* Bertol.)

**DOI:** 10.3390/plants9101339

**Published:** 2020-10-10

**Authors:** Hani Al-Ahmad

**Affiliations:** Department of Biology and Biotechnology, Faculty of Science, An-Najah National University, Nablus P.O. Box 7, Palestine; alahmad@najah.edu

**Keywords:** conservation, gibberellic acid, in vitro seed germination, Mediterranean, propagation

## Abstract

The establishment of an efficient in vitro propagation system for the conservation of the Mediterranean *Mandragora autumnalis* is highly desirable due to its scarcity, besides its potential medicinal and pharmacological properties. In a separate unpublished study, this species has proved to be resistant to laboratory plant regeneration from vegetative tissue cultures; therefore, an alternative decoated seed (i.e., endosperm enclosed the zygotic embryo) germination approach was conducted in this study. Pre-cold treatment of *M. autumnalis* seeds, removal of seed coats, and exogenous application of gibberellic acid (GA_3_) promoted in vitro seed germination and seedling emergence. In two separate experiments, approximately 10–27% of the germinated decoated seeds developed healthy seedlings within two weeks, compared to the non-germinated intact seeds of the potting soil controls. After 72 days, the highest rates of healthy seedlings development (67.4 and 69.4%) achieved in the in vitro decoated seed cultures supplemented with 60 and 100 mg/L GA_3_, respectively, compared to only 25% seedlings emergence rate of the in vitro cultures devoid of GA_3_, and 44.2% of the soil controls. The in vitro developed plants were healthy, survived transplantation conditions, and, significantly, grew faster, formed on average more than the double number of true leaves and shoot fresh weight (*p* ≤ 0.05), 90% more fresh weight of root system (*p* ≤ 0.05), and ultimately more than the double gross fresh weight (*p* ≤ 0.05) than that of the in vivo developed plants of the soil controls. Such in vitro seed germination approaches would be favorable due to the higher capacity of uniform seedling establishment year-round under lab-controlled conditions, facilitating proliferation and conservation of rare and threatened species, and providing fresh and axenic plant materials required for downstream studies such as those associated with leaf-derived protoplasts and genetic transformations.

## 1. Introduction

**Figure 1:**
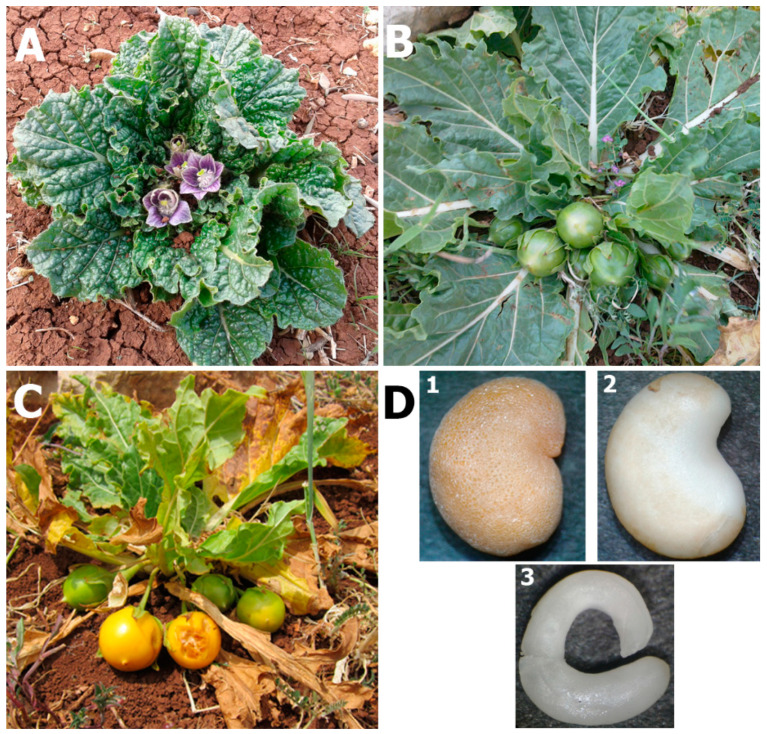
General growth features of the Mediterranean wild mandrake (*Mandragora autumnalis* Bertol.) plant. (**A**) a plant started to bloom in mid-January; (**B**) apple-shaped immature fruits in late March; (**C**) mature orange fruits in late April; (**D**) intact dry seed; (**1**) decoated seed after mechanical removal of seed coat; (**2**) an intact viable embryo isolated from decoated seed (**3**). Seed pictures were taken with the aid of a stereomicroscope (Labomed, Luxeo 4D digital stereozoom microscope™, 20× eyepieces, and 2× objective lens, Los Angeles, CA, USA).

The plant genus *Mandragora*, also known as mandrake, belongs to the nightshade family Solanaceae and comprises six species, which are distributed through Mediterranean regions to the Himalayas [1]. In the Mediterranean region, two natural species are found, *Mandragora officinalis* var. vernalis (*Mandragora officinarum*) with white flowers, and *Mandragora officinalis* var. autumnalis (*Mandragora autumnalis* Bertol.) with blue–violet flowers [2,3]. *M. autumnalis* is native to the woodlands, shrublands, and the meadows in the Middle East, Southern Europe, and North Africa. It is a stemless rosette-forming perennial herb with forked, thick, and long tuberous taproot, from which oblong–ovate leaves directly emerge. *M. autumnalis* blooming takes place in late autumn–early winter, with fertile flowers emerging on short peduncles from the center of the rosette [2] (Figure 1A). Its fruit is similar to a small apple (Figure 1B), and ripe fruits have a yellow–orange color with a pleasant scent and bearing hard-brownish kidney-shaped seeds (Figure 1C,D).

From ancient times, the mysterious *Mandragora* herb has cultural and medicinal values. It was believed to have magic, hallucinogenic, and poisonous properties [1,4,5]. It was also used as a potent pain killer, narcotic, and sedative [6], and its different parts (roots, leaves, and fruits) were used by practitioners and herbalists in traditional healing practices for the treatment of various illnesses, sexual weakness, and inflammations [7,8]. Recent phytochemical analyses of this herb have resulted with the isolation and identification of more than one hundred natural compounds, including alkanes, lipids, volatile oils, pigments, coumarins, phenols, and alkaloids [9,10,11,12]. These natural compounds have been reported to possess a variety of biological properties, including antimicrobial, antiviral, anti-inflammatory, antidiabetic, antioxidant, and enzyme inhibitory activities [13,14]. The scarce resources of wild *Mandragora* plant material due to limited seasonal-availability and its restricted geographical distribution would probably confine further investigations and essential phyto-studies at a global level.

In addition to the cultural and medicinal values, propagation and conservation of rare wild plants are important and urgent tasks in habitats where they are declining, such as in the West Bank of Palestine. Due to the in-place political conflict, the fragmented natural habitats cannot be properly monitored, neither protected nor managed. This has led to the deterioration of biodiversity, including scarce wild medicinally and culturally valuable herbs. In addition, negative impacts of climate change, drought, overgrazing, and unplanned human activities further worsened biodiversity. Therefore, proliferation and conservation of endemic species are mandated. In this scene, distribution records, population size data, and conservation measures are not available for the *M. autumnalis* [15], and it was reported as a critically endangered species in Jordan, a neighboring country of the West Bank of Palestine [16]. The growing knowledge of in vitro plant propagation and cryopreservation strategies, including in vitro seed culture, is becoming crucial towards defining potential policies and actions that help conserve wild species that are rare, endangered, or of special cultural, medicinal, economic, or ecological value [17,18,19]. The integration between traditional methods such as in situ conservation and seed storage, and advanced biotechnologies such as rapid in vitro plant propagation and multiplication, can provide successful solutions, especially when plant resources are limited.

In a long-term approach of concerted efforts, our intensive attempts towards in vitro vegetative propagation of *M. autumnalis* have been unsuccessful, as the plant has proved resistant to laboratory regeneration of whole plants from tissue cultures (unpublished data). The diverse explants that were excised from roots, leaves, flower buds, opened flowers, and immature fruits exhibited recalcitrance towards entire plant regeneration, either through common approaches of direct and indirect organogenesis or via somatic embryogenesis. Despite the viable calli obtained from immature fruits, the cells performed no morphogenetic potential, which obstructed our task towards in vitro regeneration of *M. autumnalis* plants (unpublished data).

The proliferation of *Mandragora* through seed germination would represent another option. Although it is propagated naturally through seed germination, still seed dormancy and lack of synchrony in germination will limit the rate of in vivo seed germination and subsequent plant abundance. Therefore, the main goal of this study was to establish an applicable and fast propagation protocol of wild *M. autumnalis* that can be performed at any time under controlled and pathogen-free growth conditions. In this respect, a rapid propagation approach through in vitro germination of decoated mature seeds was successful, and thus, recommended for both proliferation and conservation policies of this recalcitrant rare wild species.

## 2. Results and Discussion

### 2.1. Lifespan and Phenology of Wild Mandrake (Mandragora autumnalis Bertol.) Plants

Based on prolonged personal observations, the periodic vegetative growing season (~6 months) of the visited wild mandrake (*M. autumnalis* Bertol.) plants started early December, when first leaves sprouted from the underground tuberous taproot. The onset of flowering spanned from early January till mid- or late February based on annual rainfall frequency and temperature variability. Plants fertility was scored by ~13 flowers/plant on average (a range of 0–46 flowers/plant). Immature green fruits observed from mid-February up to mid-April with a range of ~0–19 fruits per plant (2.3 fruits/plant on average). Noticeably, some plants with many large leaves formed no flowers and thus zero seed set. Lately, yellow–orange ripe fruits enclosed 18–61 mature seeds per fruit (44 seeds/fruit on average) were observed after mid-April (Figure 1, Table 1). The dimensions of intact seeds (dry mature seeds with their hard outercoats (testa) maintained untouched), dry weight of intact seeds, as well as net weight of decoated seeds, are presented in Table 2.

### 2.2. Seed Viability vs. Periodic Storage Conditions

**Figure 2:**
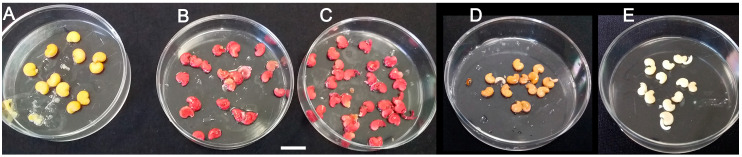
Testing viability of wild *M. autumnalis* seeds based on the tetrazolium (TZ) assay. (**A**) intact seeds remained unstained due to seed coat permeability resistance as proven with the downstream 1% methylene blue permeability assay (Figure 3); (**B**) seeds stored for 2.5 or (**C**) 3.5 years showed almost 100% viability as endosperms of decoated seeds turned red after staining treatments; (**D**) negative controls of heat-killed decoated seeds incubated for 1 h at 100 °C before TZ treatment remained unstained; (**E**) negative controls of non-treated decoated seeds. Scale size bar = 1 cm.

Surface-sterilized seeds that were stored either at 22–25 °C or 4–5 °C for up to 3.5 years, expressed 100% viability when assayed with the standard tetrazolium (TZ) viability test (Figure 2). Due to rigid seed coats, the intact seeds (Figure 2A) were impermeable to the TZ aqueous solution as the isolated endosperms from stain-treated intact seeds remained unstained (whitish). However, viable decoated seeds with active cellular dehydrogenases turned carmine red (Figure 2B,C) due to formazan precipitation [20]. The negative controls of heat-killed decoated seeds treated for 1 h incubation at 100 °C did not turn red (Figure 2D). Therefore, it is obvious that seed viability was not influenced by the long-term storage under dry/cold lab conditions. Consistently, testing seed viability with the tetrazolium salt was also performed efficiently in assaying wild type, mutated, as well as transgenic seeds of other plant species [20,21].

### 2.3. Water Permeability of M. autumnalis Seed Coats

**Figure 3:**
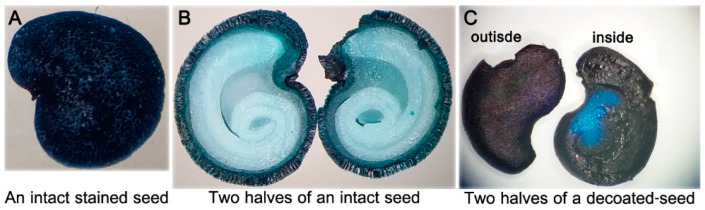
Testing water permeability potential of wild *M. autumnalis* seeds with the methylene blue assay. (**A**,**B**) intact seeds tested monthly for up to five months showed almost no penetration of the blue stain into the enclosed tissues of the endosperm and embryo; (**C**) seed coat removal resulted in fast and complete staining of the endosperm and most of the embryo within 6 days. Photos of non-stained intact seed and decoated seed, and of an isolated zygotic embryo are shown in Figure 1D above.

Supporting the above observations with the TZ staining viability-assay, intact seeds immersed in 1% methylene blue aqueous solution for up to five months were also dye-impermeable. Checked monthly, merely the outer layer of seed coats got densely blue-colored (Figure 3A), but not the enclosed whitish endosperm tissues and the zygotic embryos (Figure 3B). In contrast, the absence of seed coats of the decoated seeds permitted deep dark-blue staining of the endosperms and the embryos within 6 days (Figure 3C). Consistent results were obtained with methylene blue-treated intact vs. decoated seeds of *Ardisia crenata* [22]. This indicates that rigid seed coat of *M. autumnalis* performed physical barrier against water permeability, and thus prevented imbibition. Contrariwise, its removal would promote water uptake, which is important to initiate biochemical changes towards germination completion [22,23]. This particular concern was further empirically investigated in the afterward decoated seed germination tests.

### 2.4. Intact Mature Seed Germination and Seedling Emergence: Preliminary Tests

Artificial seed germination after specific pre/post-treatments could be one of the solutions to overcome natural seed germination constraints, which could arise due to multiple factors including seed dormancy [24]. It is thought that gibberellic acid (GA) treatment and light can both promote seed germination [25,26,27]. Therefore, in the present study, the potential of *M. autumnalis* seed germination was tested by culturing seeds on enriched seed germination media (SGM) supplemented with various levels of GA_3_. The cultures were subsequently maintained under periodic 16 hrs light, expectantly, to promote in vitro seed germination.

The preliminary results obtained from the current study revealed that intact viable mature seeds (Figure 1D, Table 2) showed recalcitrance towards germination on full-strength Murashige and Skoog medium free of GA_3_ (1X MSO) alone, or on 1X MS supplemented with various levels of GA_3_ (SGM). The in vivo germination of intact seeds was blocked despite culturing fifty seeds on monthly refreshed 1X MSO for 1 year, and of another fifty seeds tested on SGM including 0, 20, 30, 60, and 100 mg/L GA_3_ for 5 months. The mentioned seed germination trials were conducted under controlled growth chamber incubation conditions. Additionally, a 3-day pulse treatment of intact seeds with 500 mg/L GA_3_ under 100 rpm continuous shaking prior to seed culturing on SGM containing 10–50 mg/L GA_3_ failed to promote seed germination within 4 months. Here, only one (2%) normal seedling emerged after 4.5 months. In contrast, the removal of *M. autumnalis* seed coats showed a faster and higher rate of in vitro seed germination and seedling development as described later in this paper. The beneficial effects of seed coat removal on early germination have been reported among other plant species, for instance *Ardisia crenata* [22], *Mangifera indica* [28], and *Prunus yedoensis* [29].

### 2.5. Intact Seed Storage Conditions vs. Decoated Seed Germinability

A wide range of endogenous and exogenous factors participate in the control of seed dormancy and subsequent germination, with an enormous variation of levels and order of seed responses to these forces [25,29,30,31,32]. Cold stratification treatment is generally preferable for seeds of wild species to help to break seed dormancy, and to overcome endogenous seed germination inhibitors [22,33,34,35], and thus, was tested in this study. Jointly, pre-cold treatment at 4–5 °C and exogenous supplements of 30–100 mg/L GA_3_ evoked the germination rate of the in vitro cultured decoated seeds and the subsequent seedlings development over that of seeds kept at room temperature (22–25 °C), as well as over the intact seeds that were directly sown in potting soil and maintained under glasshouse conditions (Table 3). For instance, 55% and 64% of pre-cold treated seeds developed healthy seedlings when were cultured decoated on SGM supplemented with 30 and 100 mg/L GA_3_, respectively, compared to only 44% and 43% seedlings developed from decoated seeds derived from intact seeds that were stored at room temperature, and also cultured on SGM plus 30 and 100 mg/L GA_3_, respectively.

### 2.6. Removal of Seed Coats and Exogenous Application of Gibberellic acid Promote In Vitro Seed Germination and Subsequent Seedling Emergence

**Figure 4:**
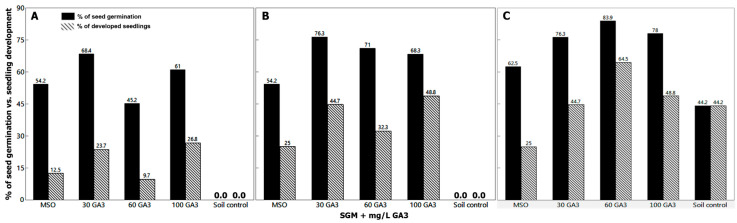
(Exp. I in 2016/2017). Percentage of *M. autumnalis* seed germination and of fully developed healthy seedlings within (**A**) 14 days; (**B**) 18 days; (**C**) 72 days of the in vitro cultured decoated seeds (*n* = 24–41) vs. intact seeds sown in potting soil (soil control), (*n* = 43). The survival frequency of the transplanted plantlets (*n* = 29) into potting soil was 100% as they reached maturity under the glasshouse conditions (Table 4 and Table 5). SGM: Seed germination medium. The intact seeds were stored at 4–5 °C.

**Figure 5:**
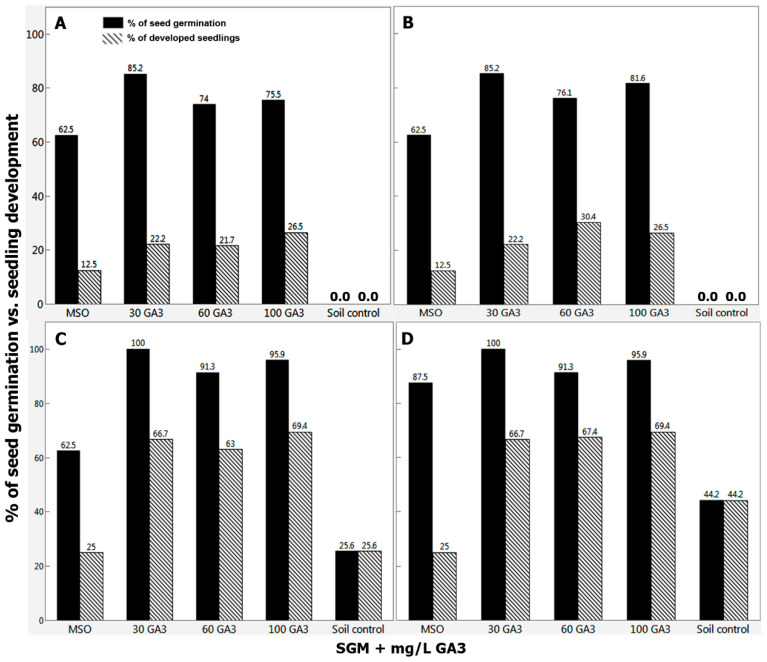
(Exp. II in 2019/2020). Percentage of *M. autumnalis* seed germination and of fully developed healthy seedlings within (**A**) 14 days; (**B**) 18 days; (**C**) 30 days; (**D**) 72 days of the in vitro cultured decoated seeds (*n* = 16–49) vs. intact seeds sown in potting soil (soil control), (*n* = 43). The intact seeds were stored at 4–5 °C.

Two primary distinctive and internally replicated experiments were conducted in the years 2016/2017 (Exp. I) and 2019/2020 (Exp. II) in order to estimate the frequencies of seed germination and normal seedlings development vs. time period and level of GA_3_ treatments. Initial in vitro decoated seed germination was observed during the first week, then reached about 45–68% (Exp. I; Figure 4A) and about 62–85% (Exp. II; Figure 5A) seed germination within 2 weeks. Consequently, healthy seedlings emerged during the second week (approx. 10–27% in Exp. I; Figure 4A, and 12–26% in Exp. II, Figure 5A) in almost a directly proportional manner to the supplemented GA_3_ levels, compared to null seed germination of intact seeds sown in parallel in the potting soil controls (Figure 4 and Figure 5). After one month, ~26% of the intact seeds sown in potting soil controls, as well as 25% of the germinated decoated seeds (~63%) of the MSO cultures developed normal seedlings, compared to ~63–69% healthy seedlings development from the germinated decoated seeds (91–100%) on SGM supplemented with 30–100 mg/L GA_3_ (Figure 5C). Therefore, the artificial culturing environment and seed coat removal, in addition to the exogenous administration of gibberellic acid, were all superior in the promotion of fast seed germination, which ultimately led to a higher rate of seedlings emergence, in a relatively short period of time compared to the soil controls of intact seed germination.

Lastly, when evaluated after 72 days, the highest rates of healthy seedlings development (67.4 and 69.4%) achieved in the in vitro decoated seed cultures supplemented with 60 and 100 mg/L GA_3_, respectively (Figure 5D), compared to only 44.2% seedlings emergence of the soil controls (Figure 4C and Figure 5D). In addition, although the decoated seeds germination rate of the in vitro MSO cultures devoid of GA_3_ elevated to 87.5% (Figure 5D), only 25% of them developed healthy seedlings (Figure 4C and Figure 5D) compared to that of the SGM cultures with added GA_3_. This fact raises again the beneficial effect of the GA_3_ in seed germination and subsequent emergence of healthy seedlings. Hence, in vitro germination of decoated seed is advantageous and would be favorable since higher rates of faster and uniform seed germination and seedling establishment were accomplished and could be employed year-round under lab-controlled conditions. On the contrary, soil controls were obviously influenced by the annual cultivation date. Seeds sown in late autumn (mimicking natural sprout time) emerged firstly after 3 weeks, with a maximum seed germination of ~44% within 72 days (Figure 4C and Figure 5D). However, seeds sown in a separate experiment in August 2019 showed a distinctive delay in germination rate, as the first seedling emerged from the soil after 3 months, with a maximum seed germination rate of 78% after 4.5 months. This is obvious since natural seed dormancy and lack of uniform seed germination are among the common inherent properties of wild plant species. In that manner, seed germination is physiologically timed to avoid unfavorable seasonal conditions that inversely influence subsequent plant establishment, growth, fitness, and survival [30,36].

In addition, classification systems have been established in order to discriminate between the distinct classes of seed dormancy, as various types, levels, and combinations of such classes are evident among many plant species [30,36,37,38]. Physical dormancy is mediated by water-impermeable testa cell layers of the seed coat [39], where physiological dormancy is induced by the phytohormone abscisic acid (ABA) produced in the endosperm. Seed dormancy release and germination are promoted by the key hormone gibberellic acid [31,40], which counteracts the role of ABA [25,30,32,40]. In seeds with coat dormancy (physical), mechanical or acid scarification of resistant seed coats can break dormancy. Examples: *Melilotus* and *Trigonella* (Fabaceae) [38,41] and *Sphaeralcea munroana* (Malvaceae) [42]. In this study, it is proposed that a combination of both physical and physiological seed dormancy influenced the temporal rate of intact seed germination of *M. autumnalis*, which were overcome by the mechanical seed coat removal in addition to the exogenous application of GA_3_. Such class of combinational dormancy has also been reported among other plant genera such as *Geranium*, *Trifolium*, *Malva* [36,37], and *Opuntia* spp. [43]. Although physiological dormancy is the most abundant dormancy type that can be broken once in response to adequate environmental signals, it seems that its combination with physical dormancy has an evolutionary adaptive advantage aim to protect such seeds from detrimental biological and physical agents from the surrounding environment, so ensure seed longevity and persistence [36]. The herein presented case of *Mandragora* could be a good example, as most seeds remained viable after long dry/cold lab storage, and performed efficient germinability, suggesting the presence of a permanent soil seed bank in the life cycle of this wild species.

### 2.7. Growth Features of the In Vitro Established M. autumnalis Plantlets Grown on SGM Cultures

**Figure 6:**
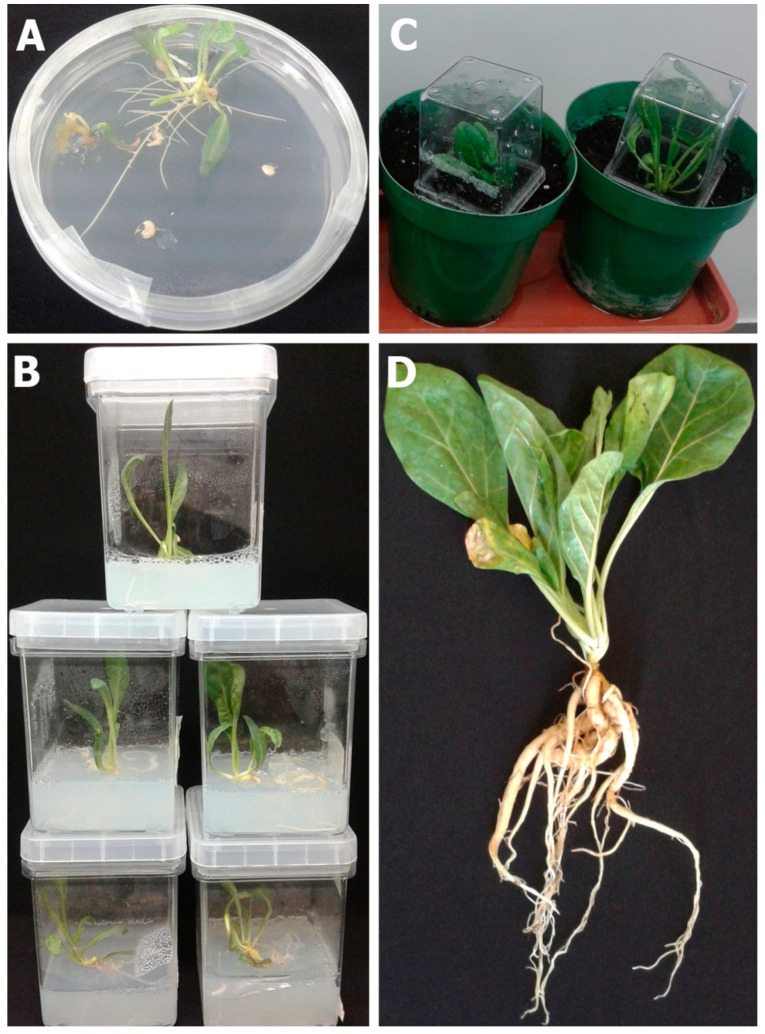
Development of healthy *M. autumnalis* plants through in vitro seed germination. (**A**) Representative normal seedling emerged from decoated seed cultured on SGM included 30 mg/L GA_3_; (**B**) representative emerged seedlings transferred to Magenta^®^ jars containing 1X MSO medium to continue normal growth; (**C**) transplanted plantlets were grown in soil under the glasshouse conditions to reach maturity (**D**).

Vegetative growth parameters were measured after 5 and 10 weeks of the in vitro developed plantlets established on the SGM in comparison to those that emerged from intact seeds sown simultaneously in the potting soil controls (Table 4). The establishment of the in vitro plantlets (Figure 6A,B) was significantly faster (*p* ≤ 0.05), as they reached the third true leaf stage in 5 weeks before the emergence of any seedling from the soil-sown seeds. In 10 weeks, when the cultured seedlings almost developed the sixth true leaf, most of the in vivo developed seedlings were almost at the first leaf stage (Table 4). Representative individual plantlets were then transplanted separately into 1-L pots, were acclimatized (Figure 6C), and allowed to continue their growth to maturity under the glasshouse conditions (Figure 6D), where further evaluations of growth performance were accomplished.

### 2.8. Growth Features of the In Vitro Established M. autumnalis Plants Transplanted into Potting Soil and Grown under Glasshouse Conditions

All the transplanted plants survived the transplantation process and the glasshouse growth conditions, and their growth features were further evaluated in comparison to the look-like plants of soil controls (Table 5). After 5 months of the in vitro culturing of decoated seeds on various SGM (=10 weeks after plants transplantation), the in vitro developed plants were healthy and significantly grew faster, formed on average more than the double number of true leaves (*p* ≤ 0.05), more than double the shoot fresh weight (*p* ≤ 0.05), 90% more fresh weight of root system (*p* ≤ 0.05), and ultimately more than the double of total plant fresh weight (*p* ≤ 0.05) than that of the 5 months-old in vivo developed plants of soil controls (Table 5). Measuring the plant shoot-height was not applicable to this rosette-type plant species. Therefore, the easily applied in vitro culturing conditions were efficient in generation and propagation of normal and healthy *Mandragora* plants after the removal of seed coats and the exogenous application of gibberellic acid.

## 3. Materials and Methods

### 3.1. Collection of Mature Seeds

Mature seeds of *M. autumnalis* were obtained from ripe yellow–orange fruits that were collected during late April and May (2016–2019) from the rare wild plants in the Mediterranean meadows and shrublands in the West Bank of Palestine. The dried seeds were stored either at 22–25 °C or 4–5 °C.

### 3.2. Surface Sterilization and Decoating of the Mature Seeds

Seeds were disinfected through surface-sterilization in 70% (*v*/*v*) ethanol for 2 min, then in full-strength commercial bleach [3% (a. i.) sodium hypochlorite] for 30 min with intervening vigorous shaking by vortex and washings with sterile distilled water, followed by final three rinses with sterile water (3 min per rinse) with intervening vigorous shaking to get rid of bleach remains. The sterilized seeds were then subjected to overnight stable imbibition treatment in distilled water to facilitate subsequent manual seed coat removal, as well as to promote embryo expansion and germination. The manual removal of the hard seed coats was accomplished using sterilized forceps and sharp scalpel without destructing the internal endosperms that enfold the zygotic embryos (termed as decoated seeds all over this paper) (Figure 1D).

### 3.3. Seed Viability Testing

Seed viability was initially tested based on the tetrazolium (TZ) assay described by Verma and Majee [20]. Intact seeds previously stored for up to 3.5 years either at 22–25 °C or 4–5 °C were firstly subjected to surface sterilization treatment. Then, representative samples (each of 25 seeds) of intact seeds vs. decoated seeds were soaked in 1% aqueous solution of 2, 3, 5-triphenyl tetrazolium chloride (Alfa Aesar, Heysham, UK) and incubated for 24 h at 30 °C in darkness. Finally, the seeds were washed with distilled water, gently dried with a paper towel, and seed viability was evaluated. Heat-killed decoated seeds treated for 1 h incubation at 100 °C were used as negative controls (Figure 2).

### 3.4. Water Permeability of Seed Coats

The potential of water permeability through *M. autumnalis* rigid seed coats was further investigated using a 1% methylene blue aqueous solution (Sigma, St. Louis, MO, USA) [43]. Intact seeds vs. decoated seeds were immersed in the methylene blue staining solution for up to 5 months and checked at monthly intervals. The seeds were washed with water, gently dried with a paper towel, cut into two halves with a sharp, clean blade, and then observed under a stereomicroscope (Labomed, Luxeo 4D digital stereozoom microscope™, 20× eyepieces, and 2× objective lens, Los Angeles, CA, USA) as described in Tezuka et al. [22], (Figure 3).

### 3.5. In Vitro Seed Germination Conditions

The decoated seeds were aseptically cultured on the SGM listed in Table 3. Surface-disinfected intact seeds were also cultured on equivalent SGM and used as control cultures to test the effectiveness of seed coat removal on seed germination and subsequent plantlets-development. In parallel, natural seed germination was also tested by direct sowing of intact seeds in potting soil as in vivo controls for the in vitro seed germination tests. The full strength (1X) basal SGM medium was prepared by mixing Murashige and Skoog (MS) [44] basal salts including vitamins according to manufacturer’s instructions (Duchefa Biochemie, Haarlem, The Netherlands) with 3% (*w*/*v*) sucrose, 100 mg/L myoinositol (Sigma, St. Louis, MO, USA) and 0.7% (*w*/*v*) Phyto Agar^®^ (Duchefa Biochemie, Haarlem, The Netherlands) as a solidifying agent. The pH of the MS medium was adjusted to 5.8 before autoclaving at 121 °C and 15 psi pressure for 20 min. When the MS medium cooled to 45 °C; selected levels of filter-sterilized GA_3_ (Alfa Aesar, Heysham, UK) were applied to SGM before being poured in Petri-dishes. In parallel, 1X MS medium free of GA_3_ (MSO) was prepared for hormone-free negative controls. All primary seed germination treatments were internally replicated and tested twice in late autumns (the natural sprout growth season of *M. autumnalis*) of years 2016/2017 (Exp. I) and 2019/2020 (Exp. II). The frequencies of initial seed germination vs. full development of healthy seedlings were recorded accordingly. Seedlings were considered healthy only if they vigorously developed two green cotyledons, a hypocotyl, a branched taproot, and ultimately formed normal plantlets. Therefore, seeds that initiated germination but failed to develop healthy seedlings were excluded from the seedlings frequency data.

### 3.6. Controlled Maintenance Conditions of Cultured Plant Materials

To physically stimulate seed germination, the cultures of both intact seeds and the decoated seeds were incubated in a standard growth chamber that maintained a continuous cycle of cool white light (80 μmoles m^−2^ s^−1^) for 16 h and 8 h of darkness, 37% relative humidity, and 25 ± 2 °C. Emerged seedlings on SGM (Figure 6A) were transferred aseptically into Magenta^®^ jars (Sigma-Aldrich) containing 1X MSO medium in order to grow and to form plantlets (Figure 6B).

### 3.7. Plantlets Transplantation and Acclimatization

For plant hardening and maturation, well-established plantlets were removed from the agar medium, the roots were washed gently with tap water, and the plantlets were transplanted into 1-L pots commercial potting soil containing enriched peat mixture (Maxifiori, Kallithea, Greece). The plantlets were immediately covered with plastic bags to maintain high humidity and transferred to a growth chamber that maintained 25 ± 2 °C and 16 h white light of 80 µmoles m^−2^ s^−1^ and 8 h of darkness for 2 weeks for hardening. The potted plants were thereafter transferred to the glasshouse to grow to maturity.

### 3.8. Statistical Analysis

The percent frequencies of seed germination and developed seedlings were presented using JMP^®^ software (Version 15; SAS Institute Inc., Cary, NC, USA, 2019). The plant growth parameters were presented as the average ± standard error (SE) and analyzed using the Minitab-17^®^ software (Minitab 17 Statistical Software, Minitab, Inc, State College, PA, USA) by one-way analysis of variance (ANOVA) Fisher’s multiple comparison tests. Probability levels were considered to be statistically significant at *p* ≤ 0.05. Differences were not considered to be statistically significant at *p* > 0.05.

## 4. Conclusions

An efficient approach towards in vitro establishment of normally developed and healthy plants was feasible for the rare wild species *Mandragora autumnalis* Bertol. Cold stratification and exogenous application of 100 mg/L gibberellic acid-GA_3_ enhanced the rate of in vitro germination of *Mandragora* decoated seeds up to 96%, of which 64% developed healthy seedlings that survived the transplantation conditions and grew normally to maturity. The procedures developed in this study would provide insights for conservation strategies for other rare and endangered wild plants. The procedures could also be adapted for in vitro axenic shoot/root biomass propagation that can be available at any time of the year for many beneficial downstream applications such as the production of secondary metabolites for medical purposes, genetic transformation, domestication trends, and protoplast isolation.

## Figures and Tables

**Table 1:** Growth productivity attributes of wild *Mandragora autumnalis* Bertol. plants visited in six natural locations (means ± SE).

Number of Leaves/Plant [Range]	Number of Flowers/Plant [Range]	Number of Immature Fruits/Plant [Range]	Number of Mature Seeds/Ripe Fruit [Range]
14.4 ± 1.2 [3–35] (*n* = 40)	13.5 ± 3.1 [0–46] (*n* = 19)	2.3 ± 0.5 [0–19] (*n* = 58)	44 ± 1.5 [18–61] (*n* = 46)

**Table 2:** Parametric characterization of mature seeds of *M. autumnalis*. (means ± SE).

Intact Seed Length (mm)	Intact Seed Width (mm)	Intact Seed Weight (mg)	Decoated Seed Weight (mg)
5.9 ± 0.1 (*n* = 21)	4.4 ± 0.1 (*n* = 21)	36.7 ± 0.9 (*n* = 50)	19.1 ± 0.8 (*n* = 21)

**Table 3:** Effect of storage temperature and gibberellic acid (GA_3_) levels on the germination potential of *M. autumnalis* decoated seed cultures vs. intact seeds directly sown in potting soil *.

Pre-Storage Temp. (°C) of Intact Seeds	Decoated Seeds on SGM + mg/L GA_3_	Number of Cultured Seeds	Number of Developed Seedlings (%)
Cold (4–5)	MSO	25	7 (28.0)
	30	49	27 (55.1)
	60	63	42 (66.7)
	100	67	43 (64.2)
Soil controls of intact seeds	-	22	10 (45.5)
Room temp. (22–25)	MSO	15	3 (20.0)
	30	16	7 (43.8)
	60	14	9 (64.3)
	100	23	10 (43.5)
Soil controls of intact seeds	-	21	9 (42.9)

* Observations were recorded after 72 days of culturing decoated seeds on the mentioned media. Seed viability of representative decoated seeds was 100% as confirmed by the TZ assay discussed earlier in this study. Intact seeds of soil controls were sown in late autumn. SGM: seed germination medium.

**Table 4:** Growth performance of *M. autumnalis* plantlets developed from decoated seed cultures vs. intact seeds directly sown in potting soil *.

Origin of Plantlets	Number of True Leaves/5 Weeks-Old Plantlet	Number of True Leaves/10 Weeks-Old Plantlet	Taproot Length (cm)/10 Weeks-Old Plantlet	Total Fresh Weight/10 Weeks-Old Plantlet (g)
In vitro decoated seeds germinated in SGM cultures	3.3 ± 0.2 ^a^	5.9 ± 0.7 ^a^	12.4 ± 0.5 ^a^	3.22 ± 0.23 ^a^
In vivo intact seeds germinated in potting soil	0.0 ± 0.0 ^b^	1.4 ± 0.2 ^b^	9.7 ± 1.2 ^b^	2.74 ± 0.20 ^a^

* Data were expressed as means ± SE. Means followed by a different letter are significantly different at *p* ≤ 0.05 based on Fisher’s multiple comparison test. N = 25–29.

**Table 5:** Growth features of five months-old in vitro vs. in vivo developed *M. autumnalis* plants *.

Origin of Plants	Number of True Leaves/Plant	Taproot Length (cm)/Plant	Fresh Weight of Shoot/Plant (g)	Fresh Weight of Root/Plant (g)	Fresh Weight of The Whole Plant (g)
In vitro decoated seeds germinated in SGM cultures	7.1 ± 0.7 ^a^ (*n* = 12)	18.8 ± 1.3 ^a^ (*n* = 11)	8.84 ± 1.57 ^a^ (*n* = 11)	11.65 ± 1.98 ^a^ (*n* = 11)	20.49 ± 3.50 ^a^ (*n* = 11)
In vivo intact seeds germinated in potting soil	2.9 ± 0.4 ^b^ (*n* = 14)	15.4 ± 1.8 ^a^ (*n* = 9)	3.72 ± 0.79 ^b^ (*n* = 9)	6.07 ± 1.11 ^b^ (*n* = 9)	9.79 ± 1.82 ^b^ (*n* = 9)

* Age of plants was calculated from the first day of the in vitro culturing of decoated seed on various SGM vs. the date when intact seeds were sown directly into potting soil. Data are expressed as means ± SE. Means followed by a different letter are significantly different at *p* ≤ 0.05 based on Fisher’s multiple comparison test.
